# Identification and Characterization of Mechanism of Action of P61-E7, a Novel Phosphine Catalysis-Based Inhibitor of Geranylgeranyltransferase-I

**DOI:** 10.1371/journal.pone.0026135

**Published:** 2011-10-18

**Authors:** Lai N. Chan, Hannah D. G. Fiji, Masaru Watanabe, Ohyun Kwon, Fuyuhiko Tamanoi

**Affiliations:** 1 Department of Microbiology, Immunology and Molecular Genetics, University of California, Los Angeles, California, United States of America; 2 Molecular Biology Institute, University of California, Los Angeles, California, United States of America; 3 Department of Chemistry and Biochemistry, University of California, Los Angeles, California, United States of America; National Taiwan University Hospital, Taiwan

## Abstract

Small molecule inhibitors of protein geranylgeranyltransferase-I (GGTase-I) provide a promising type of anticancer drugs. Here, we first report the identification of a novel tetrahydropyridine scaffold compound, P61-E7, and define effects of this compound on pancreatic cancer cells. P61-E7 was identified from a library of allenoate-derived compounds made through phosphine-catalyzed annulation reactions. P61-E7 inhibits protein geranylgeranylation and blocks membrane association of geranylgeranylated proteins. P61-E7 is effective at inhibiting both cell proliferation and cell cycle progression, and it induces high p21^CIP1/WAF1^ level in human cancer cells. P61-E7 also increases p27^Kip1^ protein level and inhibits phosphorylation of p27^Kip1^ on Thr187. We also report that P61-E7 treatment of Panc-1 cells causes cell rounding, disrupts actin cytoskeleton organization, abolishes focal adhesion assembly and inhibits anchorage independent growth. Because the cellular effects observed pointed to the involvement of RhoA, a geranylgeranylated small GTPase protein shown to influence a number of cellular processes including actin stress fiber organization, cell adhesion and cell proliferation, we have evaluated the significance of the inhibition of RhoA geranylgeranylation on the cellular effects of inhibitors of GGTase-I (GGTIs). Stable expression of farnesylated RhoA mutant (RhoA-F) results in partial resistance to the anti-proliferative effect of P61-E7 and prevents induction of p21^CIP1/WAF1^ and p27^Kip1^ by P61-E7 in Panc-1 cells. Moreover, stable expression of RhoA-F rescues Panc-1 cells from cell rounding and inhibition of focal adhesion formation caused by P61-E7. Taken together, these findings suggest that P61-E7 is a promising GGTI compound and that RhoA is an important target of P61-E7 in Panc-1 pancreatic cancer cells.

## Introduction

Proteins such as the Rho family G-proteins are posttranslationally modified by the addition of a geranylgeranyl isoprenoid [1]. The isoprenoid modification is important for membrane association and functions of these proteins. Recent studies have highlighted the significance of protein geranylgeranylation in human cancers. First, it has been shown that a number of geranylgeranylated proteins play important roles in tumorigenesis and metastasis [2]–[6]. Second, characterization of GGTase-I-deficient cells showed that the inhibition of GGTase-I leads to proliferation inhibition and accumulation of p21^CIP1/WAF1^, pointing to the significance of GGTase-I in cell proliferation and cell cycle progression [7]. Furthermore, conditional knockout of the β-subunit of GGTase-I results in the inhibition of lung tumor growth and increased survival of mice expressing oncogenic K-ras [7]. Thus, inhibition of protein geranylgeranylation is a promising approach for developing anticancer drugs, and inhibitors of GGTase-I (GGTIs) are currently undergoing preclinical studies. The results obtained are consistent with the idea that GGTIs disrupt oncogenic and tumor survival pathways, inhibit proliferation and anchorage-independent growth, and induce apoptosis [8]–[12].

A variety of approaches were taken to develop GGTI compounds. Peptidomimetic GGTI compounds were derived from the peptide bearing the C-terminal CAAL (cysteine followed by two aliphatic amino acids and the C-terminal residue is leucine or phenylalanine) motif, a sequence recognized by GGTase-I [11], [13]–[16]. The first nonpeptidomimetic inhibitor, GGTI-DU40, was identified via high-throughput screening of a compound library [17]. More recently, quantitative structure-activity relationship (QSAR) models for GGTIs have been developed and used to carry out virtual screen of more than 9 million commercially available compounds. This resulted in the identification of seven compounds with novel scaffolds [18]. Our approach [19], [20] was to construct a library of allenoate-derived small molecules based on phosphine-catalyzed annulation reactions that produce diverse compounds such as dihydropyrroles [21], [22], tetrahydropyridines [23]–[25], bicyclic succinimides, dioxanylidenes [26], α-pyrones [27], dihydropyrones [28], [29], and cyclohexenes [30]. Initial GGTI compounds identified by screening a 171-compound pilot library were then used to synthesize derivatives with increased potency. Screening the 4288 compounds enabled us to identify two types of novel GGTI compounds: one group with a dihydropyrrole ring as its core scaffold and the other group with a tetrahydropyridine ring as its core scaffold [19], [20]. In our previous study, we reported that derivatization of a carboxylic acid emanating from the dihydropyrrole ring of one of the GGTI compounds dramatically improves their cellular activity [20]. The improved GGTI, P61-A6, inhibits proliferation of a variety of human cancer cell lines, and causes G_1_ cell cycle arrest and induction of p21^CIP1/WAF1^ [20]. Our study with P61-A6 using a human pancreatic cancer xenograft model in mice showed that the GGTI exhibited prolonged blood circulation time and significant suppression of tumor growth [12].

In this study, we focused on P3-E5, a GGTI compound with a tetrahydropyridine ring [20], and modified it with an l-leucine methylester to replace the free acid. This led to the synthesis of GGTI P61-E7. Compound P61-E7 selectively inhibits protein geranylgeranlyation in cells with higher potency than P61-A6 and caused accumulation of both RhoA and RalA, which are known substrates of GGTase-I, in the cytosolic fractions. Furthermore, compared to P61-A6, P61-E7 exhibits improved potency to inhibit both cell proliferation and cell cycle progression and inducing p21^CIP1/WAF1^ level. In addition, compound P61-E7 increases p27^Kip1^ level and inhibits phosphorylation of p27^Kip1^ (T187). P61-E7 also blocks anchorage-independent growth and disrupts cell morphology of Panc-1.

We also examined the mechanism of action of P61-E7 in pancreatic cancer cells. This is important because the cellular effects observed pointed to the involvement of RhoA, a geranylgeranylated small GTPase shown to mediate a number of cellular processes such as actin stress fiber organization, cell adhesion and cell proliferation, the significance of the inhibition of RhoA geranylgeranylation on GGTI-mediated cellular effects was evaluated. We showed that stable expression of farnesylated RhoA mutant (RhoA-F) prevents induction of p21^CIP1/WAF1^ and p27^Kip1^ by P61-E7 in Panc-1 and promotes partial resistance to the anti-proliferative effect of the GGTI compound. Furthermore, stable expression of RhoA-F rescues Panc-1 cells from cell rounding and inhibition of focal adhesion formation following P61-E7 treatment. Taken together, our findings show that P61-E7 provides a novel addition to the range of GGTI compounds that are in development as anticancer drugs and that RhoA is an important target of the GGTI compound in Panc-1 pancreatic cancer cells.

## Materials and Methods

### Compounds

**Figure 1:**
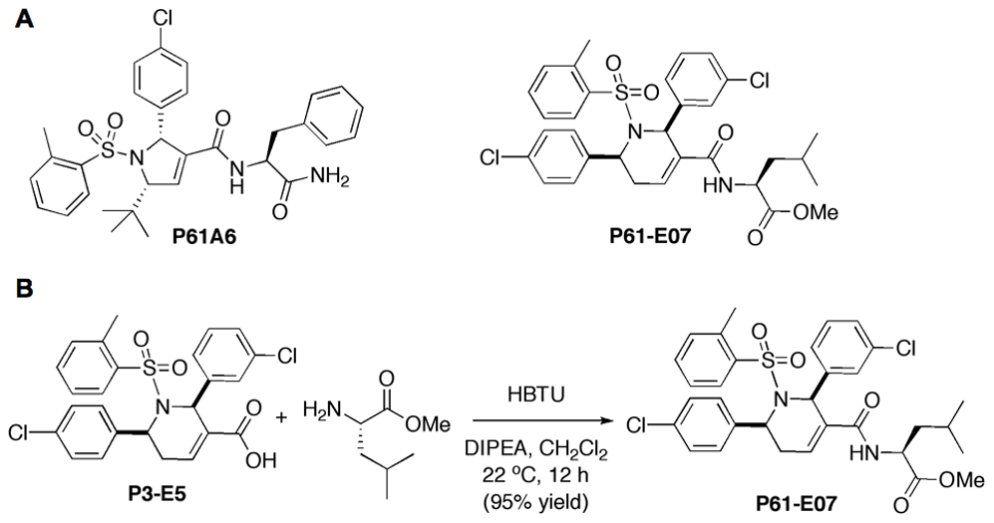
Structures of P61-A6 and P61-E7 and synthesis of P61-E7 from P3-E5. (A) Structures of compounds P61-A6 and P61-E7. (B) Treatment of P3-E5 with l-leucine methyl ester, *O*-benzotriazole-*N,N,N′,N′*-tetramethyluronium hexafluorophosphate (HBTU) and *N,N*-diisopropylethylamine (DIPEA) in dichloromethane provides P61-E7 in 95% isolated yield.

The allenoate-derived compound library was synthesized as described in our previous publications [19], [20]. Briefly, GGTI P61-E7 (Fig. 1A) was obtained by coupling P3-E5, a representative tetrahydropyridine carboxylic acid hit compound [20], with l-leucine methyl ester, where the free acid is converted to a l-leucine amido methyl ester (Fig. 1B). A 20 mM stock solution of P61-E7 in dimethyl sulfoxide (DMSO) was kept at −20°C until use. GGTI-298 was purchased from Sigma-Aldrich (St. Louis, MO). Farnesyltransferase inhibitor (FTI) BMS-225975 [31] was kindly provided by Dr. Veeraswamy Manne (Bristol-Myers Squibb Co.).

### Plasmids

We used 3xHA-RhoA pcDNA3.1 expression construct (UMR cDNA Resource Center, Rolla, MO) as template DNA for site-directed mutagenesis PCR driven by Pfu Ultra® High-Fidelity DNA Polymerase (Aligent Technologies, Wilmington, DE) according to the manufacturer's instructions with the following primers: Farnesylated RhoA-CLVS was generated using F (forward primer), 5′AGAAAAAATCTGGTTGCCTTGTCTCGTGACTCGAGTCTAG-3′, and R (reverse primer), 5′-CTAGACTCGAGTCACGAGACAAGGCAACCAGATTTTTTCT-3′.

### Cell Lines and Cell Cultures

MiaPaCa-2 (ATCC, Rockville, MD), NIH3T3 (ATCC, Rockville, MD) and Panc-1 (ATCC, Rockville, MD) cells were maintained in Dulbecco's modified Eagle's medium (DMEM; Cellgro, Herndon, VA). Jurkat (ATCC, Rockville, MD) cells were maintained in RPMI 1640 medium (Cellgro, Herndon, VA). Both media were supplemented with 10% (v/v) fetal bovine serum (FBS; HyClone, Logan, UT) and 1% penicillin/1% streptomycin stock solution (Invitrogen, Carlsbad, CA). All cells were cultured at 37°C in a humidified incubator at 5% CO_2_.

### Transfection Procedure and Creation of Stable Cell Lines

Panc-1 cells were grown to 80% confluency on 6-well plates and transfected with Lipofectamine™ 2000 (Invitrogen, Carlsbad, CA) according to the manufacturer's instructions. Briefly, 10 µl of Lipofectamine™ 2000 reagent was diluted in 250 µl of OPTI-MEM medium (Invitrogen, Carlsbad, CA) and was allowed to equilibrate at room temperature for 5 min. 4.0 µg of plasmid DNA per 250 µl of medium was suspended and allowed to complex with the diluted Lipofectamine™ 2000 for 20 min at room temperature. Transfection complexes were added to cells in serum-free DMEM without antibiotics and incubated at 37°C for 4–6 h. Medium was then changed and replaced with DMEM containing 10% FBS, with penicillin-streptomycin, and the cells were further incubated at 37°C for 48 h. To generate stable cell lines, cells were passaged at 1∶10, 1∶20, and 1∶50 dilutions into selective medium containing 800 µg/ml of Geneticin® (G418 sulfate; Invitrogen, Carlsbad, CA). Cell clones were expanded into mass culture, and expression of 3xHA-RhoA-GG and 3xHA-RhoA-F was analyzed by Western blotting using antibodies against the HA tag.

### Subcellular Fractionation

NIH3T3 cells and Panc-1 cells stably expressing 3xHA-RhoA were treated with DMSO, P61-A6, or P61-E7 for 48 h. Cells were then washed and scraped into phosphate-buffered saline (PBS) and centrifuged at 2,500 rpm for 5 min. Pellets were resuspended (10 mM HEPES/KOH at pH 7.3, 10 mM KCl, 5 mM MgCl_2_, 0.5 mM DTT, and 1× protease inhibitor mixture), incubated on ice for 30 min and homogenized. Homogenates were centrifuged at 1000×*g* for 10 min to collect the cytosolic fractions (supernatant). The remaining pellets were then resuspended in buffer containing 1% Triton X-100, 150 mM NaCl, 20 mM Tris-HCl at pH 7.5, 1 mM EDTA, and 1× protease inhibitor mixture, and centrifuged at 15, 000 rpm for 15 min to collect the membrane-containing fractions (supernatant). Na^+^/K^+^ ATPase-α and RhoGDI were used as markers for the membrane-containing fractions and the cytosolic fractions, respectively.

### Western Blotting

Cells were treated with DMSO, FTI BMS-225975, GGTI-298, P61-A6 or P61-E7 for 48 h, harvested, and lysed in lysis buffer (1% Triton X-100, 150 mM NaCl, 20 mM Tris-HCl at pH 7.5, 1 mM EDTA, and 1× protease inhibitor mixture). Proteins were then resolved by 12% or 12.5% SDS-PAGE and immunoblotted with antibodies against unprenylated form of Rap1 (U-Rap1; Santa Cruz Biotechnology, Inc.), p21^CIP1/WAF1^ (Millipore, Temecula, CA), p27^Kip1^ (rabbit, Santa Cruz Biotechnology, Inc.), phospho-p27^Kip1^ (T187) (Invitrogen, Carlsbad, CA), HDJ-2 (NeoMarkers, Fremont, CA), RhoGDI (Santa Cruz Biotechnology, Inc.), Na^+^/K^+^ATPase-α (Sigma), RalA (BD Bioscience), HA.11 (Covance) and Actin (Calbiochem). Detection was performed using peroxidase-conjugated secondary antibodies (Biorad) and Amersham ECL Plus™ Western Blotting Detection Reagents (GE Healthcare Life Sciences). Select bands were quantified using ImageJ imaging processing program (National Institutes of Health).

### Immunoprecipitation of p27^Kip1^ Protein

Panc-1 cells were lysed in lysis buffer (1% Triton X-100, 150 mM NaCl, 20 mM Tris-HCl, pH 7.5, 1 mM EDTA, and 1× protease inhibitor mixture). Lysates were incubated with p27^Kip1^ antibodies (rabbit, Santa Cruz Biotechnology, Inc.) in 50% Protein G/Sepharose beads slurry for 2 h at 4°C while rocking, then washed four times with an excess of lysis buffer. Samples were then boiled at 95°C for 10 min in 4× sodium dodecyl sulfate (SDS)-sample buffer and analyzed by Western blotting as described above.

### Cell Proliferation Assays and Cell Cycle Analyses

Effects of GGTIs on cell proliferation were examined using the CCK-8 cell counting kit (Dojindo Molecular Technologies, Kumamoto, Japan) as described previously [20]. Briefly, cells (3×10^3^) were seeded onto 96-well plates. The following day cells were treated with the appropriate inhibitor as indicated in the figure legends under low-serum conditions (0.5% FBS) for 72 hours. Cell Proliferation was calculated relative to the DMSO control. The cell cycle profiles were analyzed by flow cytometry as described previously [32].

### Fluorescence Microscopy

To examine the effects of GGTI on actin cytoskeleton and focal adhesion formation, cells were seeded on 4-well or 8-well chamber slides. The following day, cells were treated as indicated in the figure legends. Then cells were fixed in 3.7% formaldehyde-PBS and permeabilized in 0.1% Trion X-100-PBS for actin fiber and vinculin detection. Actin fibers were detected by incubation with fluorescein isothiocyanate (FITC)-labeled phalloidin or tetramethyl rhodamine isothiocyanate (TRITC)-labeled phalloidin (Sigma-Aldrich, St. Louis, MO) as indicated in figure legends. Vinculin was detected with anti-vinculin antibodies (mouse, Sigma-Aldrich, St. Louis, MO) and fluorescently conjugated anti-mouse IgG antibody (Sigma-Aldrich, St. Louis, MO). Cell images were visualized using a Zeiss Microscope (40× magnification). Images were captured using AxioVision software.

### Anchorage Independent Growth Assay

Cells were seeded at a cell density of 1000 cells/well in duplicate in 12-well culture dishes in 0.3% agar over a 0.5% bottom agar layer. Various concentrations of P61-E7 or DMSO were incubated in the top layer of cells. Cultures were re-fed and treated with the GGTI or DMSO once weekly (14 days of incubation in total). Colonies were stained with 1 mg/ml MTT (tetrazolium salt) for 1 hour and scanned.

## Results

### Synthesis of GGTI P61-E7 by the Derivatization of the Carboxyl Group on the Dihydropyrrole Ring of GGTI P3-E5

We previously reported a novel small molecule GGTI compound P61-A6 (Figure 1A) [20]. This compound was identified by screening a pilot library of allenoate-derived compounds made through phosphine catalysis for scaffolds that can be used for further derivatization. The dihydropyrrole scaffold identified was then derivatized to identify compounds with improved potency and bioavailability. A particularly useful observation was made concerning a modification of the carboxyl group off of the dihydropyrrole ring that resulted in a dramatic increase in cellular potency [19], [20]. Along the way, we succeeded in establishing a novel chemical library of >4,000 compounds through phosphine-catalyzed annulations of resin-bound allenoates.

The above initial study identified another scaffold that consists of a tetrahydropyridine ring. P3-E5, a representative tetrahydropyridine carboxylic acid compound, inhibits GGTase-I with IC_50_ value of 0.31 µM [20]. The inhibition was specific to GGTase-I, as little inhibition was observed with FTase and RabGGTase (Figure S1). In this study, we derivatized the carboxyl group of P3-E5 to generate compounds that exhibit increased cellular activity. From a series of modified compounds, we identified P61-E7 that was synthesized by coupling carboxylic acid of P3-E5 with l-leucine methyl ester to yield an amido ester P61-E7 (Figure 1B). The reaction carried out under the influence of *O*-benzotriazole-*N,N,N′,N′*-tetramethyluronium hexafluorophosphate (HBTU) and *N,N*-diisopropylethylamine (DIPEA) in chloromethane [33] was efficient with 95% yield. P61-E7 demonstrated better cellular GGTI activity than P3-E5; GI_50_ values with Jurkat cells were 3.5 µM and 20 µM for P61-E7 and P3-E5, respectively.

### GGTI P61-E7 Exhibits Improved Ability to Inhibit Geranylgeranylation in Cultured Mammalian Cells When Compared to GGTI P61-A6

**Figure 2:**
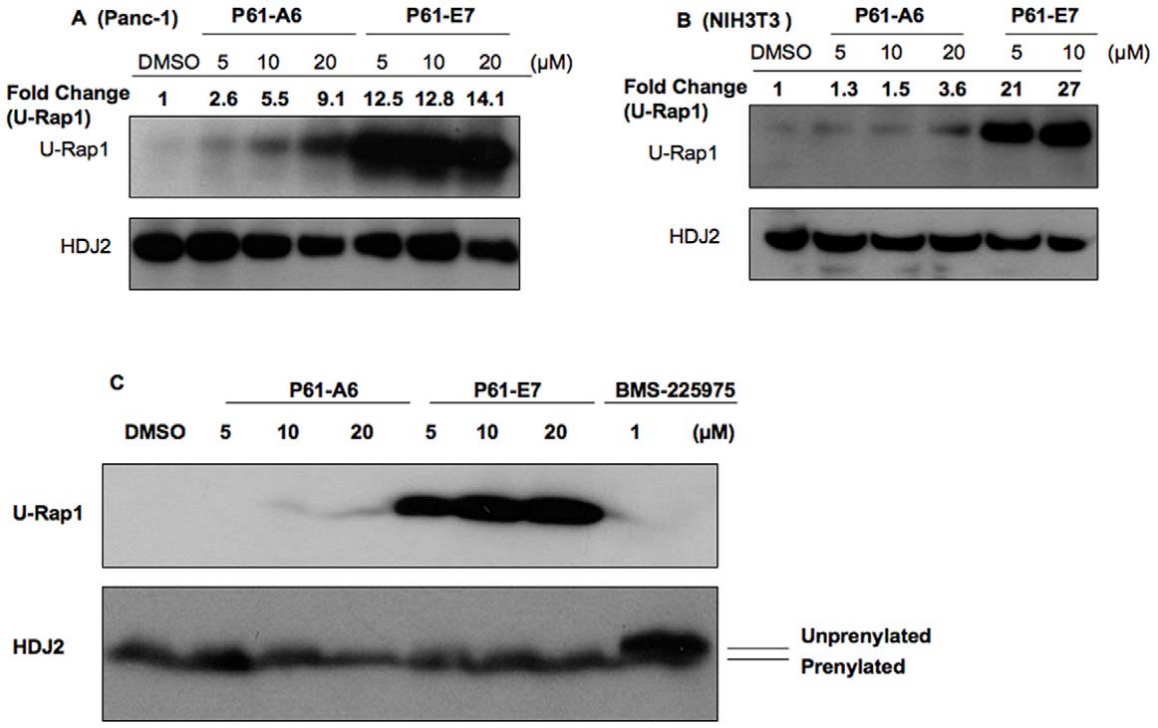
GGTI P61-E7 is a more potent inhibitor of geranylgeranylation compared with P61-A6. (A and B), P61-E7 or P61-A6 treatment inhibits Rap1 geranylgeranylation in Panc-1 (A) and NIH3T3 (B) cells. Whole cell lysates from cells treated with DMSO, P61-A6, or P61-E7 for 48 h were prepared and resolved on SDS-PAGE for immunoblotting analysis using antibodies against the unprenylated form of Rap1 (U-Rap1, upper panels, A and B), or HDJ2 (loading control, lower panels, A and B). Data shown are representative of three independent experiments. The U-Rap1 and HDJ2 bands were quantified using ImageJ. Intensities of U-Rap1 bands were normalized to their respective loading control, and the results are given above the images as fold change compared to the DMSO control. (C) Whole cell lysates from NIH3T3 cells treated with DMSO, P61-A6, P61-E7, or FTI BMS-225975 for 48 h were prepared and resolved on SDS-PAGE for immunoblotting analysis using antibodies against the unprenylated form of Rap1 (U-Rap1, upper panel), or HDJ2 (lower panel). Data shown are representative of three independent experiments.

P61-E7 exhibits increased potency to inhibit protein geranylgeranylation in cells (Figure 2A and 2B). In the first assay, we assessed inhibition of protein geranylgeranylation by using an antibody that specifically detects unprenylated form of Rap1 [34]. Rap1 is a Ras-like GTPase that plays a role in cadherin-based cell adhesion [34], and is a known substrate of GGTase-I. Panc-1 (Figure 2A) and NIH3T3 cells (Figure 2B) were treated with DMSO, P61-A6, or P61-E7 for 48 hours. Treatment with either P61-A6 or P61-E7 led to the accumulation of unprenylated Rap1 in a dose-dependent manner in both Panc-1 (Figure 2A) and NIH3T3 (Figure 2B) cells. P61-E7 treatment resulted in the inhibition of protein geranylgeranylation at much lower concentrations when compared to P61-A6 in both cell lines tested (Figure 2A and 2B). For example, at 5 µM, P61-A6 increased the level of unprenylated Rap1 in Panc-1 cells by 2.6-fold when compared to DMSO, while 5 µM of P61-E7 caused the level of unprenylated Rap1 in Panc-1 cells to increase by 12.5-fold (Figure 2A). Likewise, at 5 µM, P61-A6 treatment did not significantly change the level of unprenyalted Rap1 in NIH3T3 cells compared to DMSO treatment, while 5 µM of P61-E7 resulted in 21-fold increase in unprenylated Rap1 level (Figure 2B). All these findings reflected a significant improvement in the potency of P61-E7 in inhibiting protein geranylgeranylation in cells.

Next, the specificity of P61-E7 was measured by determining its ability to inhibit farnesylation of HDJ2 which is a cochaperone of the heat-shock cognate protein 70 (hsc70) involved in protein folding and traffic [35] and a known substrate of farnesyltransferase (FTase). NIH3T3 cells were treated with various concentrations of P61-E7 (5, 10, and 20 µM) or BMS-225975, an FTase inhibitor (FTI). While treatment with BMS-225975 slowed the mobility of HDJ2 on SDS-polyacrylamide gel, no such mobility shift was observed with P61-E7 treatment even when cells were treated with 20 µM of P61-E7 (Figure 2C). These findings indicated that P61-E7 selectively inhibits protein geranylgeranylation but not farnesylation in cells.

### Treatment with P61-E7 results in increase of cytosolic localization of geranylgeranylated proteins

**Figure 3:**
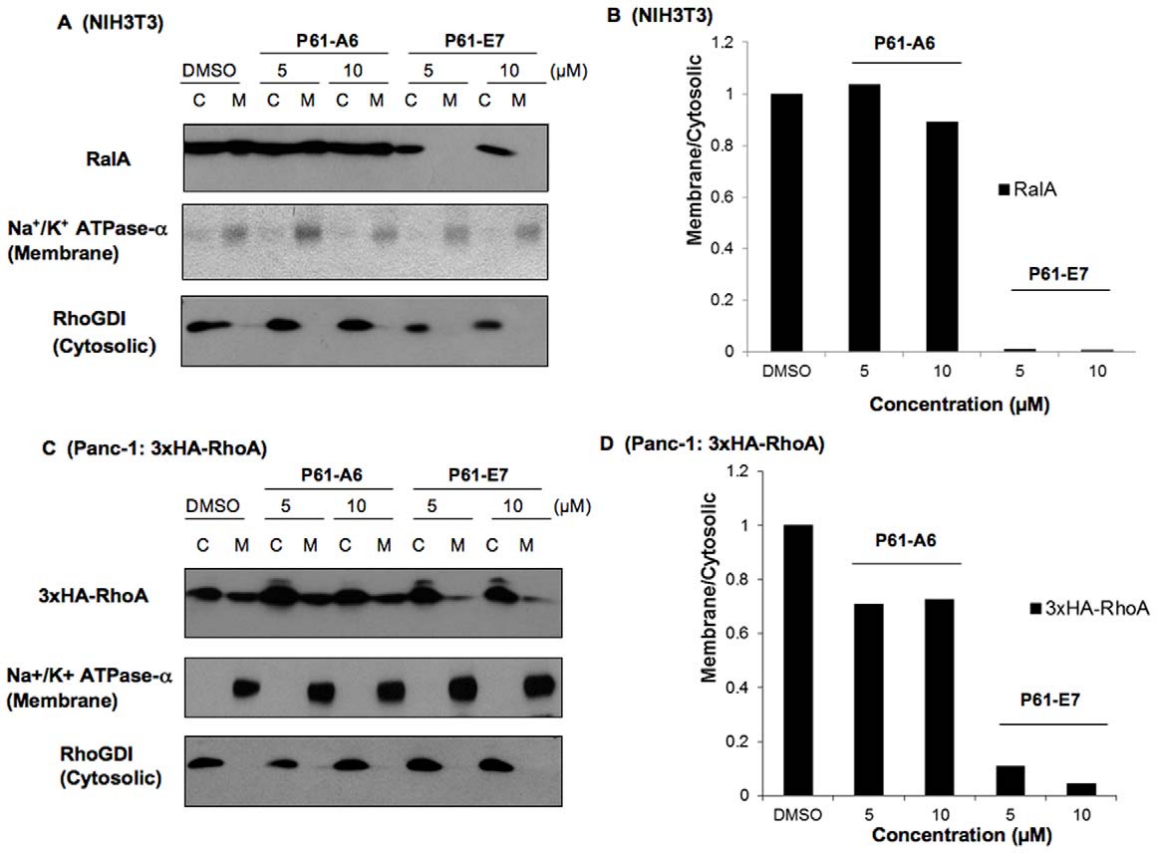
P61-E7 causes an increase of cytosolic RalA and RhoA in comparison to P61-A6. NIH3T3 cells (A,B) and Panc-1 cells stably expressing 3xHA-RhoA (C,D) were treated with DMSO, P61-A6, or P61-E7 for 48 h. Cytosolic and membrane fractions were prepared and processed for SDS-PAGE, followed by Western blotting using antibodies against RalA (A, top panel), the HA tag (C, top panel), Na^+^/K^+^ ATPase-α (A and C, middle panels) and RhoGDI (A and C, bottom panels). Data shown are representative of three independent experiments. The RalA (A, top panel), 3xHA-RhoA (C, top panel), Na^+^/K^+^ ATPase-α (A and C, middle panels) and RhoGDI (A and C, bottom panels) bands were quantified using ImageJ. Intensities of RalA and 3xHA-RhoA bands in the membrane or cytosolic fraction were normalized to their respective control for membrane or cytosolic fraction. Subcellular localization of RalA (B) and 3xHA-RhoA (D) are represented as Membrane/Cytosolic ratio, relative to the DMSO controls (which were set at a value of one).

Because prenylation is required for geranylgeranylated proteins to be associated with cellular membranes, we assessed whether P61-E7 treatment inhibits membrane association of proteins such as RhoA and RalA, known substrates of GGTase-I. NIH3T3 cells (Figure 3A and 3B) and Panc-1 cells stably expressing 3xHA-RhoA (Figure 3C and 3D) were treated with DMSO, P61-A6 or P61-E7, and membrane and cytosolic fractions were prepared and processed for SDS-PAGE followed by Western blotting. The Na^+^/K^+^ ATPase-α membrane marker and the RhoGDI cytosolic marker were used to confirm the separation of membrane-containing and cytosolic fractions, respectively. As shown in Figure 3, treatment with P61-E7 resulted in increases of RalA (Figure 3A and 3B) and 3xHA-RhoA (Figure 3C and 3D) in the cytosolic fractions of NIH3T3 cells and Panc-1cells stably expressing 3xHA-RhoA, respectively, while their association with the membrane fractions was decreased. Furthermore, at both concentrations tested (5 and 10 µM), P61-E7 is more effective at disrupting association of RalA (Figure 3A and 3B) and 3xHA-RhoA (Figure 3Cand 3D) with the membrane fractions when compared to P61-A6.

### P61-E7 is More Effective at Inhibiting Cell Cycle Progression, Inducing p21^CIP1/WAF1^ Protein Level and Blocking Cell Proliferation When Compared to P61-A6

**Figure 4:**
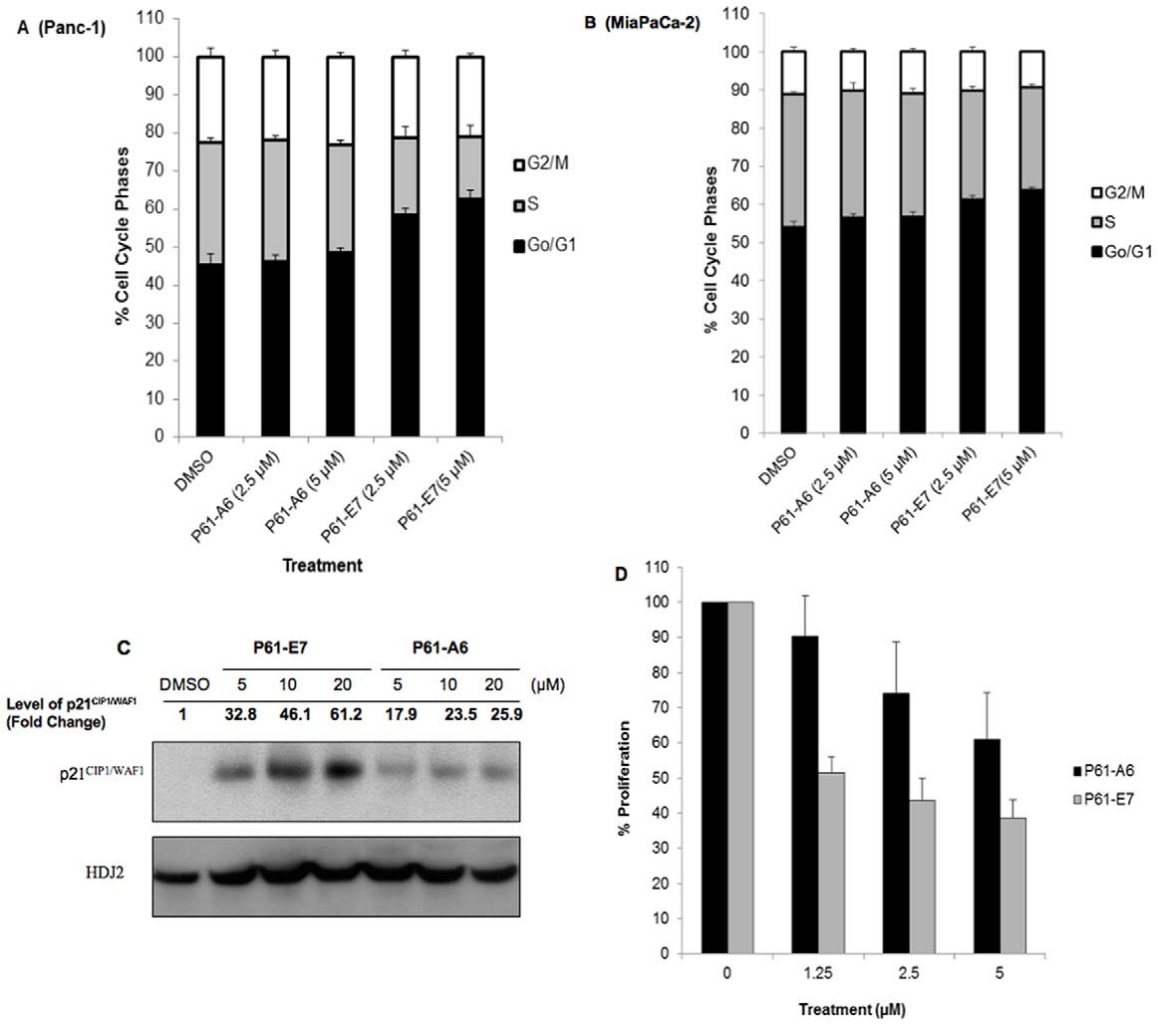
P61-E7 exhibits improved potency to inhibit cell cycle progression and cell proliferation. Panc-1 (A) and MiaPaCa-2 (B) cells were treated with DMSO, or the indicated concentrations of P61-E7 or P61-A6 for 48 h. Cell cycle profiles were monitored by flow cytometry. The percentages of cells in each phase of the cell cycle are indicated by different shades. Data shown are averages ± S.D. of three independent experiments. (C) Panc-1 cells were treated with DMSO, or various concentrations of P61-E7 or P61-A6, and whole cell lysates were collected and resolved on SDS-PAGE for immunoblotting using antibodies against p21^CIP1/WAF1^. Results shown are representative of three independent experiments. The p21^CIP1/WAF1^ and RhoGDI (loading control) bands were quantified using ImageJ. Intensities of p21^CIP1/WAF1^ bands were normalized to their respective loading control, and the results are given above the images as fold change compared to the DMSO control. (D) Panc-1 cells were treated with DMSO, P61-A6, or P61-E7 under low-serum conditions (0.5% FBS) for 72 hours. Cell Proliferation relative to the DMSO control (100%) is plotted. Data shown are averages of two independent experiments, each in quadruplet.

As shown in Figure 4, treatment of Panc-1 (Figure 4A) and MiaPaCa-2 (Figure 4B) cells with either P61-A6 or P61-E7 for 48 h caused dose-dependent enrichment of G_1_ phase cells, whereas the percentage of S phase cells decreased. Consistent with findings discussed above, P61-E7 is more effective at inhibiting cell cycle progression in both cell lines tested (Figure 4A and 4B). For example, following treatment with P61-A6 (5 µM), the proportion of Panc-1 cells in the G_1_ phase increased from 45.1%±3.1% (DMSO) to 48.4%±1.4%, whereas the proportion of Panc-1 cells in the G_1_ phase increased to 62.7%±2.2% following treatment with P61-E7 (5 µM) (Figure 4A). Likewise, at 5 µM, P61-A6 treatment caused an increase in proportion of MiaPaCa-2 cells in the G_1_ phase by 2.6%±1.3% in comparison to DMSO treatment, while treatment of MiaPaCa-2 cells with 5 µM of P61-E7 increased the proportion of cells in the G_1_ phase by 9.7%±0.67% when compared to DMSO treatment (Figure 4B).

The accumulation of G_1_ phase cells was associated with an induction of proteins involved in the regulation of cell cycle. Both P61-A6 and P61-E7 induced p21^CIP1/WAF1^ expression level in Panc-1 (Figure 4C) and NIH3T3 (data not shown) cells in a dose-dependent manner. Similar to observations discussed above, P61-E7 treatment resulted in a higher induction of p21^CIP1/WAF1^ level when compared to treatment with P61-A6 in both cell lines tested. For example, at 20 µM, P61-A6 induced p21^CIP1/WAF1^ level by 25.9-fold in comparison to DMSO treatment, while treatment with 20 µM of P61-E7 induced p21^CIP1/WAF1^ level by 61.2-fold (Figure 4C).

Besides inducing p21^CIP1/WAF1^ expression, P61-E7 increased p27^Kip1^ protein level in Panc-1 cells (Figure S2A). Phosphorylation of p27^Kip1^ on Thr187 by cyclin E/CDK2 results in the association of p27^Kip1^ with SCF ubiquitin ligase, targeting it for degradation. It has been shown that Cdk2 phosphorylates p27^Kip1^ on Thr187 and promotes its nuclear degradation, and that the inhibition of the phosphorylation results in nuclear accumulation of p27^Kip1^ [36]. Therefore, we investigated whether P61-E7 inhibits phosphorylation of p27^Kip1^ on Thr187 in Panc-1 cells. P61-E7 increased p27^Kip1^ protein level in Panc-1 cells (Figure S2B and S2C), while inhibiting the phosphorylation of p27^Kip1^ on Thr187 (Figure S2B).

The abilities of P61-A6 and P61-E7 to inhibit proliferation of Panc-1 cells under low-serum conditions (0.5%) were examined and compared. Consistent with findings discussed above, P61-E7 is more effective at inhibiting cell proliferation (Figure 4D). For example, at 1.25 µM, P61-A6 treatment inhibited proliferation by 9.72%±11.5% in comparison to DMSO treatment, while treatment with 1.25 µM of P61-E7 inhibited proliferation by 48.6%±4.49%. The improved potency of P61-E7 to inhibit cell proliferation when compared to P61-A6 reflects its increased ability to inhibit protein geranylgeranylation inside the cells (Figure 2).

### P61-E7 Causes Actin Cytoskeleton Disorganization, Abolishes Formation of Focal Adhesions, and Inhibits Anchorage-Independent Growth of Panc-1 Cells

**Figure 5:**
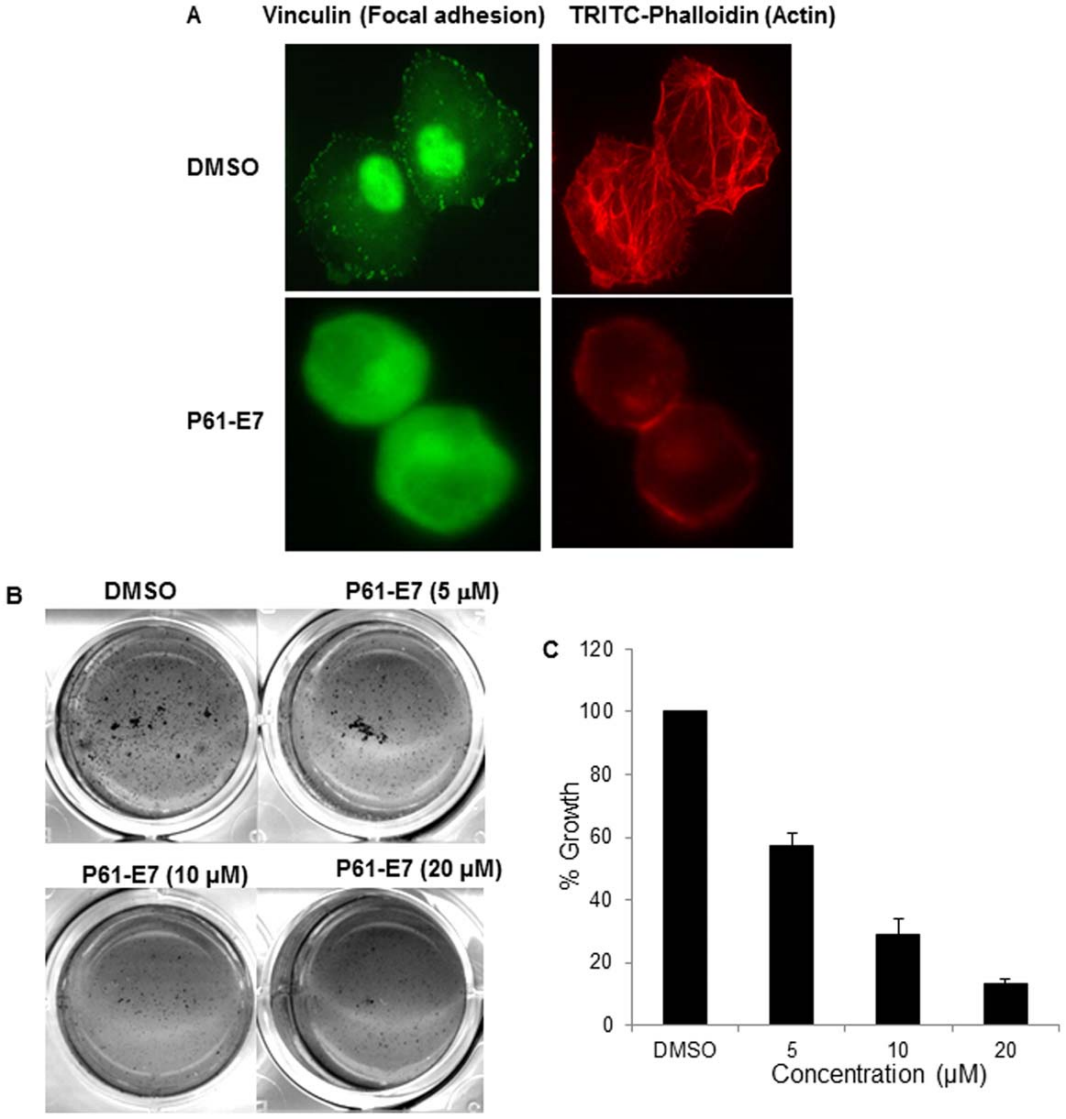
Effects of P61-E7 on cell morphology and anchorage-independent growth of Panc-1. (A) Panc-1 cells were serum-starved in the presence of DMSO or 5 µM P61-E7 for 24 h, followed by stimulation with 10% FBS in DMEM for 30 minutes. Actin fibers and focal adhesions were visualized with TRITC-phalloidin and mouse anti-vinculin followed by FITC-conjugated anti-mouse antibody, respectively. Data are representative of three independent experiments. (B) Cells were seeded at a cell density of 1000 cells/well in 12-well culture dishes in 0.3% agar over a 0.5% bottom agar layer. DMSO or various concentrations of P61-E7 were included in the top layer of cells. Cultures were re-fed and treated with the GGTI or DMSO once weekly (14 days of incubation in total). Colonies were stained with 1 mg/ml MTT for 1 hour and scanned. Shown are representative results from three independent experiments, each in duplicate. (C) P61-E6 inhibits anchorage-independent growth of Panc-1. Growth in soft agar relative to the DMSO control (100%) is plotted. Shown are the averages ± S.D. of three independent experiments, each in duplicate.

To further investigate cellular effects of P61-E7, we carried out experiments using Panc-1 cells. First, we examined the effects of P61-E7 on the cell morphology of Panc-1 cell. Previous studies have shown that GGTase-I deficiency results in disrupted actin cytoskeleton in fibroblasts [7], and that GGTI treatment causes disorganization of actin cytoskeleton and inhibits formation of vinculin-containing focal adhesions [37]. Panc-1 cells were treated with DMSO or P61-E7 and the cells were stained with phalloidin to observe actin cytoskeleton. As shown in Figure 5A, P61-E7 treatment caused disorganization of actin cytoskeleton and significant cell rounding. We next examined whether P61-E7 treatment had effects on focal adhesion. This was examined by using antibody against vinculin, one of the components of focal adhesion. As seen in Figure 5A, the treatment with P61-E7 led to the disappearance of vinculin punctate immunostaining at the periphery of Panc-1 cells. The staining became uniform throughout the cell. The GGTI treatment had no effect on vinculin expression level (data not shown). These results suggest that focal adhesion assembly was inhibited by P61-E7.

In addition, a soft agar clonogenicity assay was used to measure the effect of P61-E7 on the anchorage-independent growth of Panc-1 cells. Treatment with increasing concentrations of P61-E7 impaired the anchorage-independent growth of Panc-1 cells in a dose-dependent manner (Figure 5B and 5C). For examples, at 5 µM, P61-E7 inhibited colony formation by 42.9%±4.1%, while it inhibited colony formation by 71.2%±5.2% at 10 µM (Figure 5B).

### Stable Expression of Farnesylated RhoA in Panc-1 Cells Suppresses GGTI-induced Phenotypes

RhoA, a geranylgeranylated small GTPase, has been shown to influence a number of cellular processes including actin stress fiber organization, cell adhesion and cell proliferation. Interestingly, the cellular effects of P61-E7 observed pointed to the possible involvement of RhoA in GGTI-mediated cellular effects. First, the above results on the effects of GGTI to induce p21^CIP1/WAF1^ are consistent with previous studies that have shown that small GTPase protein RhoA (RhoA-GG) acts as a negative regulator of p21^CIP1/WAF1^ expression [38], [39]. Effects of GGTI on actin cytoskeleton also support the possibility that P61-E7 induces cellular effects by blocking geranylgeranylation of RhoA, thereby disrupting its subcellular localization and inhibiting its functions. Indeed, results from our RhoA-GTP pull-down assay indicated that P61-E7 inhibited activation of RhoA in a dose-dependent manner (Figure S3). To assess the implication of RhoA in P61-E7 induced phenotypes, we constructed Panc-1 stable cell line expressing farnesylated RhoA (RhoA-F). Using site-directed mutagenesis, a RhoA-F mutant was generated by altering the CAAX from CLVL to CLVS sequence to render RhoA a substrate of farnesyltransferase. Previous studies showed that farnesylated RhoA has the same subcellular localization as geranylgeranylated RhoA, and that both RhoA prenyl isoforms have similar effects on cell morphology, actin organization, vinculin distribution, cell proliferation and p21^CIP1/WAF1^ expression [40]. However, unlike wild-type RhoA (RhoA-GG), RhoA-F is expected to be resistant to GGTI treatment. Therefore, if P61-E7 does indeed increase p21^CIP1/WAF1^ expression by blocking geranylgeranylation of RhoA, stable expression of RhoA-F is expected to overcome P61-E7's effect on p21^CIP1/WAF1^ expression.

**Figure 6:**
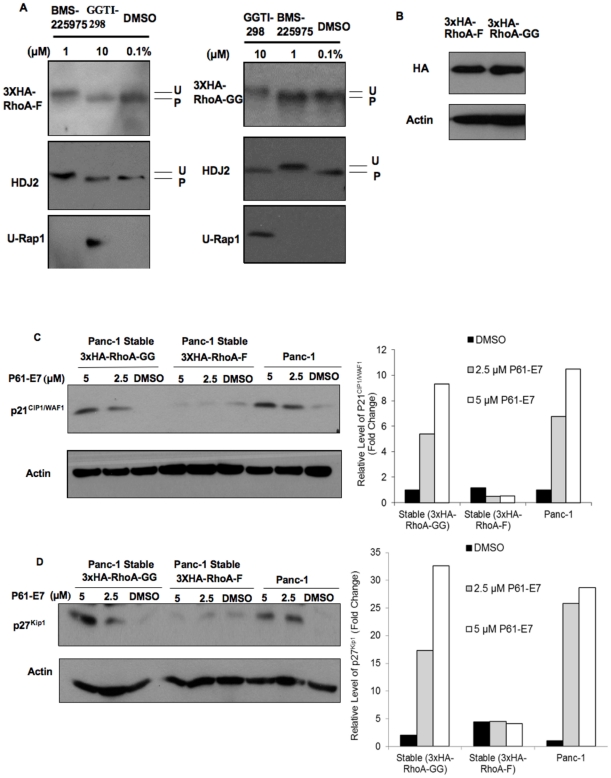
Stable expression of farnesylated RhoA prevents P61-E7-mediated increases in p21^CIP/WAF1^ and p27^Kip1^ protein levels. (A) Effects of GGTI-298 and FTI BMS-225975 on prenylation of wild-type 3xHA-RhoA (RhoA-GG, geranylgeranylated) and 3x-HA-RhoA-F (farnesylated) mutant. Panc-1 cells stably expressing 3xHA-RhoA-GG (right panels) or 3xHA-RhoA-F (left panels) were treated DMSO, BMS-225975, or GGTI-298 for 48 h. Whole cell lysates were collected and analyzed for RhoA, Rap1, and HDJ2 processing by Western blotting. (B) Expression levels of 3xHA-RhoA-F and 3xHA-RhoA-GG in Panc-1 stable cell lines used in this experiment were analyzed by immunoblotting using antibodies against the HA tag or actin (loading control). (C and D) Panc-1 cells and Panc-1 cells stably expressing 3xHA-RhoA-GG or 3xHA-RhoA-F were treated with DMSO or P61-E7 for 48 h. Whole cell lysates were collected and analyzed for protein levels of p21^CIP/WAF1^ (C, top panel), p27^Kip1^ (D, top panel), or Actin (C and D, bottom panels). Data shown are representative of three independent experiments. The p21^CIP1/WAF1^ (C, top panel), p27^Kip1^ (D, top panel), and Actin (C and D, bottom panels) bands were quantified using ImageJ. Protein Levels of p21^CIP1/WAF1^ and p27^Kip1^ are represented as relative level compared to the DMSO control.

We established populations of Panc-1 cells stably expressing either 3xHA-tagged wild-type RhoA (3xHA-RhoA-GG) or 3xHA-RhoA mutant (3xHA-RhoA-F) as described in “Materials and Methods,” and confirmed the prenylation of 3xHA-RhoA-GG and 3xHA-RhoA-F in these stable cell lines by treating them with DMSO, FTI BMS-225975, or GGTI-298 which is a commercially available GGTI. Treatment with GGTI-298 inhibited protein geranylgeranylation as indicated by the appearance of the unprenylated Rap1 band (Figure 6A, left and right panels). Moreover, as expected, GGTI-298 treatment slowed the mobility of 3xHA-RhoA-GG on SDS-PAGE, indicating geranylgeranylation of 3xHA-RhoA-GG was inhibited (Figure 6A, right panel). However, GGTI-298 did not change the mobility of 3xHA-RhoA-F protein (Figure 6A, left panel). Consistent with observations discussed above (Figure 2C), FTI BMS-225975 inhibited farnesylation of HDJ2, a known substrate of FTase, as shown by the appearance of the slower migrating form of HDJ2 on SDS-PAGE (Figure 6A, left and right panels). Furthermore, treatment with the FTI inhibited farnesylation of 3xHA-RhoA-F (Figure 6A, left panel) while it had no effect on the mobility of 3xHA-RhoA-GG (Figure 6A, right panel). We also checked the expression levels of RhoA-GG and RhoA-F in the Panc-1 stable cell lines used in these experiments. As indicated in Figure 6B, the levels of RhoA-GG and Rho-F were similar in these stable cell lines.

To investigate whether stable expression of RhoA-F prevents P61-E7 induction of p21^CIP1/WAF1^, Panc-1 cells and Panc-1 cells stably expressing either the wild-type RhoA (3xHA-RhoA-GG) or mutant RhoA (3xHA-RhoA-F) were treated with DMSO or various concentrations of P61-E7. Consistent with previous observations, increases in p21^CIP1WAF1^ protein levels were observed in Panc-1 cell and Panc-1 cells stably expressing 3xHA-RhoA-GG (Figure 6C). On the contrary, induction of p21^CIP1/WAF1^ was not observed in Panc-1 cells stably expressing 3xHA-RhoA-F (Figure 6C). These results indicated that stable expression of 3xHA-RhoA-F in Panc-1 prevented induction of p21^CIP1/WAF1^ by P61-E7.

We also found that the expression of RhoA-F prevented induction of p27^Kip1^ protein level by P61-E7. We treated Panc-1 cells and Panc-1 cells stably expressing either 3xHA-RhoA-GG or 3xHA-RhoA-F with DMSO or P61-E7. Consistent with previous observations, increases in p27^Kip1^ protein levels were observed in Panc-1 cell and Panc-1 cells stably expressing 3xHA-RhoA-GG (Figure 6D). On the other hand, P61-E7 did not increase p27^Kip1^ protein level in Panc-1 cells stably expressing 3xHA-RhoA-F (Figure 6D). These results showed that stable expression of 3xHA-RhoA-F in Panc-1 inhibited the ability of P61-E7 to increase p27^Kip1^ level.

**Figure 7:**
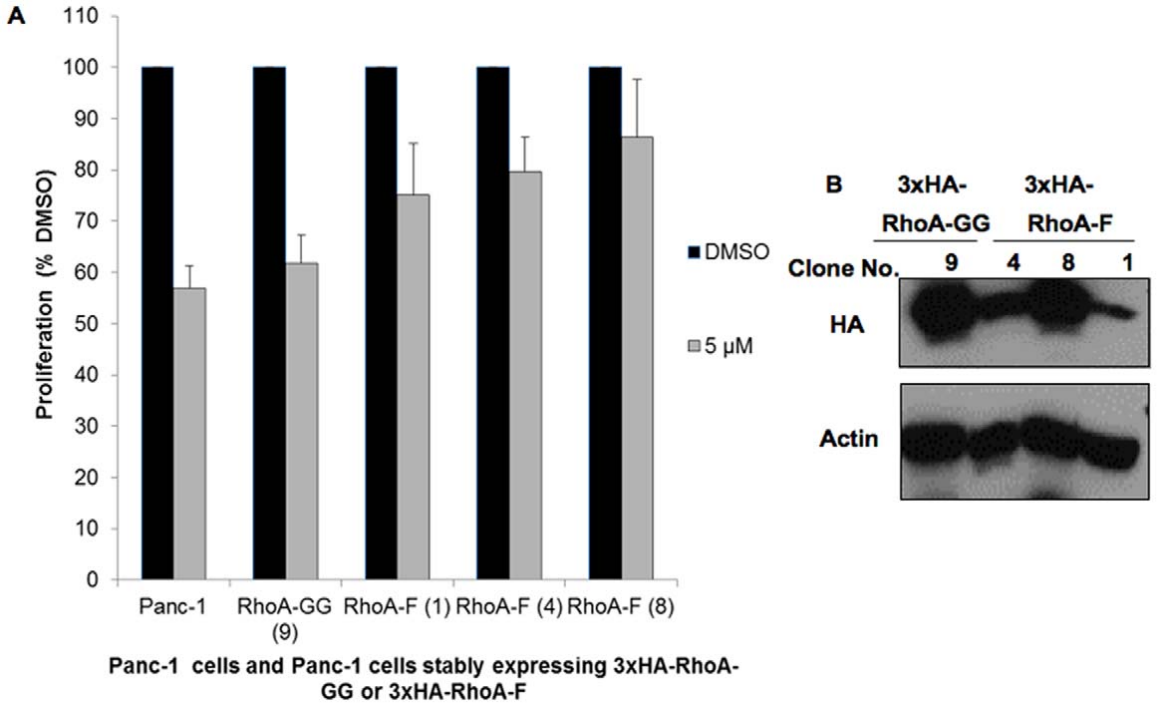
Stable expression of farnesylated RhoA promotes partial resistance to the anti-proliferative effect of P61-E7. (A) Stable expression of farnesylated RhoA (3xHA-RhoA-F) partially prevents P61-E7 inhibition of proliferation in Panc-1 cells in a manner that depends on the expression levels of 3xHA-RhoA-F. Panc-1 cells and Panc-1 stable cell lines expressing either wild-type 3xHA- RhoA-GG (clone no. 9) or RhoA-F mutant (clone no. 1, 4, or 8) were treated with DMSO or various concentrations of P61-E7 for 72 h. Cell number was determined as described in “Materials and Methods.” Cell proliferation relative to the DMSO control (100% value) is plotted. Data shown are averages ± S.D. of three independent experiments. (B) Protein levels of 3xHA-RhoA-GG (clone no. 9) and 3xHA-RhoA-F (clone no. 1, 4, or 8) in different clones of Panc-1 stable cell lines as analyzed by immunoblotting using antibodies against the HA tag or actin (loading control).

Next, we investigated whether stable expression of farnesylated RhoA confers resistance to the antiproliferative effect of P61-E7. Following treatment with DMSO or 5 µM of P61-E7, cell proliferation of Panc-1 cells, Panc-1 cells stably expressing 3xHA-RhoA-GG, and 3 different clones of Panc-1 cells stably expressing 3xHA-RhoA-F with different expression levels (Figure 7B) was measured as described previously [20]. As shown in Figure 7A, stable expression of 3xHA-RhoA-F promotes partial resistance to the anti-proliferative effect of P61-E7 in a manner that depends on its expression level in Panc-1 cells. With the highest expression level of RhoA-F, Panc-1 (3xHA-RhoA-F) Clone No. 8 showed the highest resistance to the anti-proliferative effect of P61-E7 when compared with Panc-1 (3xHA-RhoA-F) Clone No. 1 (lowest expression level) and Clone No. 4 (intermediate expression level). These findings suggested that P61-E7 inhibition of cell proliferation in Panc-1 cells is due, in part, to inhibition of RhoA geranylgeranylation and hence its function.

**Figure 8:**
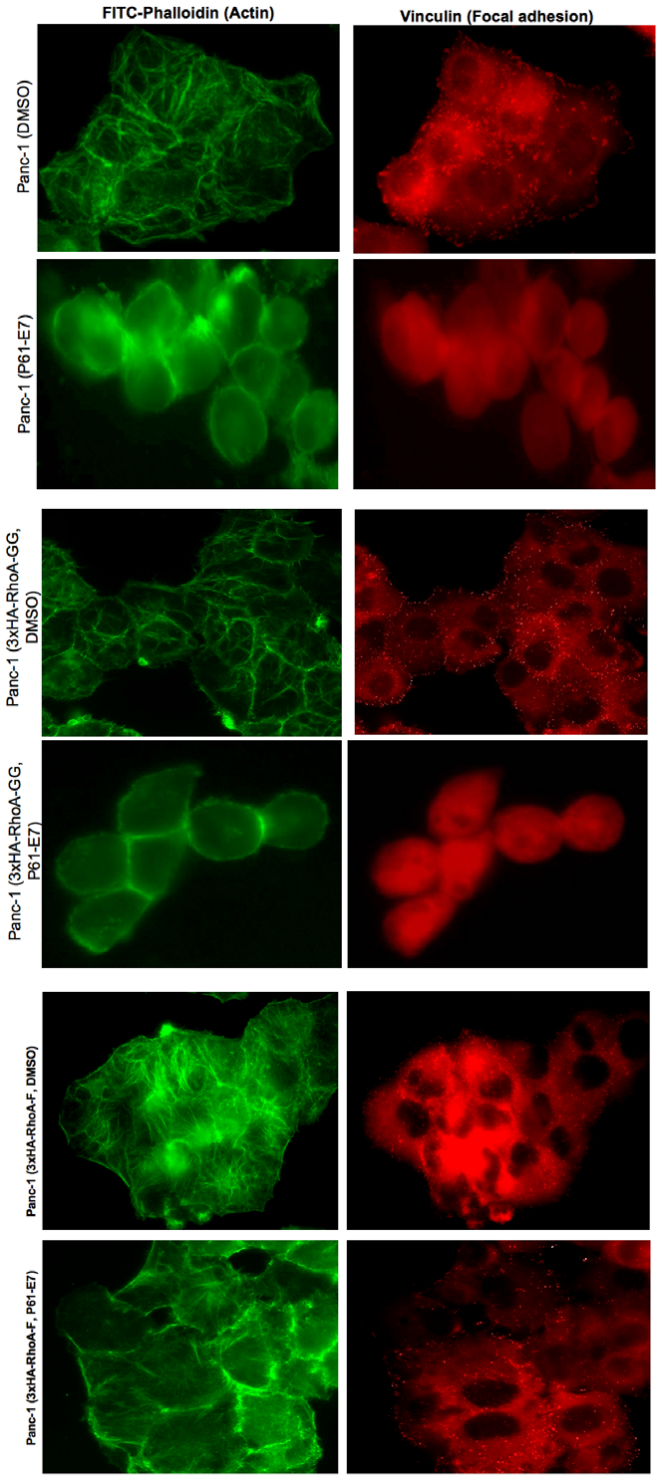
Stable expression of farnesyalted RhoA rescues cells from cell rounding and inhibition of focal adhesion assembly caused by P61-E7. Panc-1 cells, Panc-1 cells stably expressing 3xHA-RhoA-GG wild-type, and Panc-1 cells stably expressing 3xHA-RhoA-F mutant were serum-starved in the presence of DMSO or 5 µM P61-E7 for 48 h, followed by stimulation with 10% FBS in DMEM. Actin fibers and focal adhesions were visualized with FITC-phalloidin and mouse anti-vinculin followed by TRITC-conjugated anti-mouse antibody, respectively. Data are representative of three independent experiments.

We also examined whether cell morphology and actin cytoskeleton changes induced by P61-E7 can be rescued by the stable expression of 3xHA-RhoA-F in Panc-1 cells. As shown in Figure 8, Panc-1 cells expressing 3xHA-RhoA-GG responded to P61-E7, and morphological changes and actin cytoskeleton disorganization were observed. In addition, vinculin staining showed that focal adhesion formation was inhibited by the GGTI treatment. In contrast, P61-E7 failed to induce morphological changes and actin cytoskeleton changes in Panc-1 cells expressing HA-RhoA-F. Furthermore, focal adhesions were still detected even after P61-E7 treatment in this cell line. These results suggest that stable expression of RhoA-F rescues Panc-1 cells from cell rounding and inhibition of focal adhesion formation caused by P61-E7.

## Discussion

In this paper, we report successful identification of a novel GGTI compound P61-E7 that has a tetrahydropyridine scaffold. P61-E7 was derived by converting the free carboxylic acid moiety on the tetrahydropyridine ring of a parent compound P3-E5 to l-leucine amido methyl ester. P61-E7 exhibits increased potency to inhibit protein geranylgeranylation compared to our previous preclinical compound P61-A6 [12], [20]. Inhibition of geranylgeranylation was first shown by detecting the appearance of unprenylated Rap1. In addition, P61-E7 was shown to be more effective at inducing cytoplasmic accumulation of RalA and RhoA in comparison to P61-A6.

The availability of a compound with increased potency enabled us to define cellular consequences of inhibiting protein geranylgeranylation. By using two compounds with different levels of potency to inhibit geranylgeranylation (P61-E7 and P61-A6), we could look for cellular effects that correlate with the inhibition of geranylgeranylation. We examined the effects of P61-E7 on pancreatic cancer cells Panc-1. We have shown that P61-E7 causes more pronounced cell cycle block at the G_1_ phase compared with P61-A6. In addition, P61-E7 showed higher level of p21^CIP1/WAF1^ induction in comparison to P61-A6. Furthermore, P61-E7 treatment resulted in a more pronounced inhibition of cellular proliferation under low serum condition when compared to P61-A6. The excellent correlation we observed between cellular effects and increased potency to inhibit protein geranylgeranylation supports the idea that the consequences of geranylgeranylation inhibition include inhibition of cell cycle progression, induction of p21^CIP1/WAF1^ and inhibition of proliferation.

We also examined the mechanism of action of P61-E7 in this paper, and our work suggests that RhoA is a major player in GGTI-mediated effects on pancreatic cancer cells. We have shown that P61-E7 induces p27^Kip1^ level, suggesting that the treatment with GGTI results in the increases of both p21^CIP1/WAF1^ and p27^Kip1^ in Panc-1 cells. The mechanism of induction, however, appears to differ between the two Cdk inhibitors. In the case of p21^CIP1/WAF1^, it appears that the induction is regulated at the transcriptional level, as we have shown that GGTI induces expression of p21-reporter construct [20]. On the other hand, GGTI effect on p27^Kip1^is associated with the inhibition of phosphorylation of Thr187. Phosphorylation of p27^Kip1^ (T187) results in the association of p27^Kip1^ with SCF ubiquitin ligase, targeting p27^Kip1^ for degradation [36]. Because P61-E7 inhibits phosphorylation of p27^Kip1^ (T187), it is possible that P61-E7 prevents degradation of p27^Kip1^, causing accumulation of p27^Kip1^ in the nucleus in Panc-1 cells.

The above results on the effects of GGTI on Cdk inhibitors raise the possibility that the GGTI effects are mediated by RhoA. First, it is known that RhoA acts as a negative regulator of p21^CIP1/WAF1^ expression [38], [39], [41]. Second, RhoA is shown to be essential for the degradation of p27^Kip1^ [42]–[44] and that RhoA stimulates p27^Kip1^ degradation through its regulation of cyclin E/CDK2 activity [43]. Further support for the idea that many of the GGTI effects we observed with Panc-1 cells are due to the inhibition of RhoA function comes from additional effects of P61-E7 we observed. We found that P61-E7 treatment results in cell rounding, actin cytoskeleton disorganization and inhibition of focal adhesion formation.

To evaluate the possibility that GGTI targets RhoA functions, we have established stable cell lines of Panc-1 expressing a mutated form of RhoA (RhoA-F). RhoA-F has the CAAL (CLVL) box changed to CAAX (CLVS) so that the mutant protein is farnesylated instead of being geranylgeranylated. Characterization of these stable cell lines showed that the expression of RhoA-F reversed the ability of GGTI to induce p21^CIP1/WAF1^ and p27^Kip1^. Stable expression of RhoA-F rescued cells from cell rounding and focal adhesion inhibition caused by P61-E7. Finally, we observed partial rescue of GGTI-induced proliferation inhibition and this rescue correlated with the expression levels of RhoA-F. These results suggest that many of the GGTI effects are due to the inhibition of RhoA functions. Our results on the rescue of proliferation inhibition by the expression of RhoA-F is consistent with the previous report that the expression of farnesylatable RhoA and Cdc42 restores proliferation of GGTase-I deficient fibroblasts expressing oncogenic K-Ras [7]. There are other proteins implicated in the GGTI effects. One type of proteins is Ral suggested to be involved in the GGTI effects of MiaPaca-2 pancreatic cancer cells [4]. It is possible that the Ral proteins are involved in other aspects of GGTI effects such as tumor growth. In addition, it has been suggested that Rac1and Rac3 proteins are involved in the GGTI effects on fibroblast cells [45]. Further work is needed to express mutant forms of these proteins that can bypass geranylgeranylation to gain complete understanding of the mechanism of GGTI effects.

## Supporting Information

Figure S1 **Effects of P3-E5 on the enzymatic activities of GGTase-I, RabGGTase, and FTase.** GGTase-I and FTase activities were determined by following the incorporation of radiolabeled isoprenoid [^3^H]geranylgeranyl or [^3^H]farnesyl into substrate proteins. FTase or GGTase-I (50 nM) was used to initiate reactions containing 0.4 µM [^3^H]farnesyl diphosphate (FPP) (21.5 Ci/mmol, Perkin Elmer Life Sciences) or 0.5 µM [^3^H]geranylgeranyl diphosphate (GGPP) (23.0 Ci/mmol, Perkin Elmer Life Sciences) and 2 µM maltose-binding protein-tagged substrates (K-Ras4B for FTase and RhoA for GGTase-I) in 20 µl of buffer containing 50 mM Tris-HCl (pH 7.5), 10 mM MgCl_2_, 5 µM ZnCl_2_, 0.01% Triton X-100, and 1 mM dithiothreitol. Inhibitors were added at the indicated concentrations. The final DMSO concentration was 2.5% for all samples. Reactions were carried out for 10 min at 30°C. The reaction mixture was spotted onto a filter paper and treated with 10% trichloroacetic acid, followed by ethanol and acetone washing. The filter was counted using a scintillation counter. For RabGGTase assays, the reaction contained the following components in 20 µl: 0.625 µl of [^3^H]GGPP (0.7 µM), 25 nM RabGGTase, 0.6 µM REP-1, 0.6 µM purified Rab7 or Ypt1 protein, 40 mM HEPES (pH 7.5), 150 mM NaCl, 5 mM dithiothreitol, 3 mM MgCl_2_, and 0.3% CHAPS. Reactions were carried out for 20 min at 37°C, and the products were analyzed as described above for the GGTase-I reaction. Data represent the mean ± S.D. of two measurements from two independent experiments.
(TIF)

Figure S2 **Effects of P61-E7 on p27^Kip1^ protein levels.** (A) Panc-1 cells were treated with DMSO or various concentrations of P61-E7, and whole cell lysates were collected and resolved on SDS-PAGE for immunoblotting using antibodies against p27^Kip1^. Results shown are representative of three independent experiments. (B) and (C) Panc-1 cells were treated with DMSO or P61-E7 (5 µM) for 48 h. Whole cell lysates were collected and were immunoprecipitated (B) with anti-p27^Kip1^ antibodies in 50% Protein G/Sepharose beads slurry (negative control for immunoprecipitation: whole cell lysates from untreated Panc-1 cells mixed with slurry, without antibodies). (B) Immunoprecipitates were then resolved on SDS-PAGE for immunoblot analysis using phospho-27^Kip1^(T187) antibodies (B, top panel) or p27^Kip1^ antibodies (B, bottom panel). Lanes: 1, DMSO-treated; 2, P61-E7-treated; 3, No antibody control. (C) Remaining lysates (10 µg) from each sample were resolved on SDS-PAGE for immunoblotting using antibodies against p27^Kip1^ (C, top panel) or actin (C, bottom panel) to determine total p27^Kip1^ level in each input used for immunoprecipitation. Results shown are representative of three independent experiments. The RhoGDI, Actin, phospho-p27^Kip1^ and total p27^Kip1^ bands were quantified using ImageJ, and the results are given above the images as fold change compared to the DMSO control.
(TIF)

Figure S3 **P61-E7 inhibits RhoA activation in Panc-1 cells.** Panc-1 cells were transfected with 3xHA-RhoA pcDNA expression vector. Twenty-four hours after transfection, cells were serum-starved in the presence of DMSO or P61-E7 for 24 h. Then, cells were stimulated with 10% FBS in DMEM in the presence of DMSO or P61-E7 for 30 min. Whole cell lysates were collected using Mg^2+^-containing lysis buffer, and GTP-RhoA was pulled down using GST-tagged Rhotekin-RBD protein beads (Cytoskeleton) following the manufacturer's instructions. Whole cell lysates (inputs) and pull-down were resolved on SDS-PAGE for immunoblotting analysis using HA.11 antibodies to detect total 3xHA-RhoA (bottom panel) and GTP-bound 3xHA-RhoA (top panel), respectively.
(TIF)

## References

[1] Tamanoi F, Sigman DS (2001). The Enzymes. Vol. 21..

[2] Lim KH, Baines AT, Fiordalisi J, Shipitsin M, Feig LA (2005). Activation of RalA is critical for Ras-induced tumorigenesis of human cells.. Cancer Cell.

[3] Lim KH, O'Hayer K, Adam SJ, Kendall SD, Campbell PM (2006). Divergent roles for RalA and RalB in malignant growth of human pancreatic carcinoma cells.. Current Biology.

[4] Falsetti SC, Wang DA, Peng H, Carrico D, Cox AD (2007). Geranylgeranyltransferase I inhibitors target RalB to inhibit anchorage-dependent growth and induce apoptosis and RalA to inhibit anchorage-independent growth.. Mol Cell Biol.

[5] Clark EA, Golub TR, Lander ES, Hynes RO (2000). Genomic analysis of metastasis reveals an essential role for RhoC.. Nature.

[6] Harem A, Sanchez-Sweatman O, You-Ten A, Duncan G, Wakeham A (2005). RhoC is dispensable for embryogenesis and tumor initiation but essential for metastasis.. Genes Dev.

[7] Sjogren AK, Andersson KM, Liu M, Cutts BA, Karlsson C (2007). GGTase-I deficiency reduces tumor formation and improves survival in mice with K-RAS-induced lung cancer.. J Clin Invest.

[8] Dan HC, Jiang K, Coppola D, Hamilton A, Nicosia SV (2004). Phosphatidylinositol-3-OH kinase/AKT and survivin pathways as critical targets for geranylgeranyltransferase I inhibitor-induced apoptosis.. Oncogene.

[9] Miquel K, Pradines J, Sun J, Qian Y, Hamilton AD (1997). GGTI-298 induces G0-G1 block and apoptosis whereas FTI-277 causes G2-M enrichment in A549 cells.. Cancer Res.

[10] Sun J, Blaskovich MA, Knowles D, Qian Y, Ohkanda J (1999). Antitumor efficacy of a novel class of non-thiol-containing peptidomimetic inhibitors of farnesyltransferase and geranylgeranyltransferase I: combination therapy with the cytotoxic agents cisplatin, Taxol, and gemcitabine.. Cancer Res.

[11] Sun J, Ohkanda J, Coppola D, Yin H, Kothare M (2003). Geranylgeranyltransferase I inhibitor GGTI-2154 induces breast carcinoma apoptosis and tumor regression in H-Ras transgenic mice.. Cancer Res.

[12] Lu J, Chan L, Fiji HD, Dahl R, Kwon O (2009). In vivo antitumor effect of a novel inhibitor of protein geranylgeranyltransferase-I.. Mol Cancer Ther.

[13] Sebti SM, Adjei AA (2004). Farnesyltransferase Inhibitors.. Semin Oncol.

[14] Lerner EC, Qian Y, Hamilton AD, Sebti SM (1995). Disruption of oncogenic K-Ras4B processing and signaling by a potent geranylgeranyltransferase I inhibitor.. J Biol Chem.

[15] Vasudevan A, Qian Y, Vogt A, Blaskovich MA, Ohkanda J (1999). Potent, highly selective, and non-thiol inhibitors of protein geranylgeranyltransferase-I.. J Med Chem.

[16] Sun J, Qian Y, Hamilton AD, Sebti SM (1998). Both farnesyltransferase and geranylgeranyltransferase I inhibitors are required for inhibition of oncogenic K-Ras prenylation but each alone is sufficient to suppress human tumor growth in nude mouse xenografts.. Oncogene.

[17] Peterson YK, Kelly P, Weinbaum CA, Casey PJ (2006). A novel protein geranylgeranyltransferase-I inhibitor with high potency, selectivity, and cellular activity.. J Biol Chem.

[18] Peterson YK, Wang XS, Casey PJ, Tropsha A (2009). Discovery of geranylgeranyltransferase-I inhibitors with novel scaffolds by the means of quantitative structure-activity relationship modeling, virtual screening, and experimental validation.. J Med Chem.

[19] Castellano S, Fiji HD, Kinderman SS, Watanabe M, Leon P (2007). Small-molecule inhibitors of protein geranylgeranyltransferase type I.. J Am Chem Soc.

[20] Watanabe M, Fiji HD, Guo L, Chan L, Kinderman SS (2008). Inhibitors of protein geranylgeranyltransferase I and Rab geranylgeranyltransferase identified from a library of allenoate-derived compounds.. J Biol Chem.

[21] Zhu X, Henry CE, Kwon O (2005). Phosphine-catalyzed synthesis of 1,3-dioxan-4-ylidenes.. Tetrahedron.

[22] Andrews IP, Kwon O (2011). Phosphine Catalyzed [3+2] Annulation: Synthesis of Ethyl 5-(tert-Butyl)-2-phenyl-1-Tosyl-3-Pyrroline-3-Carboxylate.. Org Synth.

[23] Zhu X-F, Lan J, Kwon O (2003). An expedient phosphine-catalyzed [4 + 2] annulation: synthesis of highly functionalized tetrahydropyridines.. J Am Chem Soc.

[24] Lu K, Kwon O (2009). Phosphine-Catalyzed [4+2] Annulation: Synthesis of Ethyl 6-Phenyl-1-Tosyl-1,2,5,6-Tetrahydropyridine-3-Carboxylate.. Org Synth.

[25] Guo H, Xu Q, Kwon O (2009). Phosphine-promoted [3+3] annulations of aziridines with allenoates: facile entry into highly functionalized tetrahydropyridines.. J Am Chem Soc.

[26] Zhu X, Henry CE, Wang J, Dudding T, Kwon O (2005). Phosphine-catalyzed synthesis of 1,3-dioxan-4-ylidenes.. Org Lett.

[27] Zhu X, Schaffner A, Li RC, Kwon O (2005). Phosphine-catalyzed synthesis of 6-substituted 2-pyrones: manifestation of E/Z-isomerism in the zwitterionic intermediate.. Org Lett.

[28] Creech GS, Kwon O (2008). Alcohol-assisted phosphine catalysis: one-step syntheses of dihydropyrones from aldehydes and allenoates.. Org Lett.

[29] Creech GS, Zhu X, Fonovic B, Dudding T, Kwon O (2008). Theory-Guided Design of Bronsted Acid-Assisted Phosphine Catalysis: Synthesis of Dihydropyrones from Aldehydes and Allenoates.. Tetrahedron.

[30] Tran YS, Kwon O (2007). Phosphine-catalyzed [4+2] annulation: synthesis of cyclohexenes.. J Am Chem Soc.

[31] Gau CL, Kato-Stankiewicz J, Jiang C, Miyamoto S, Guo L (2005). Farnesyltransferase inhibitors reverse altered growth and distribution of actin filaments in Tsc-deficient cells via inhibition of both rapamycin-sensitive and –insensitive pathways.. Mol Cancer Ther.

[32] Kato-Stankiewicz J, Hakimi I, Zhi G, Zhang J, Serebriiskii I (2002). Inhibitors of Ras/Raf-1 interaction identified by two-hybrid screening revert Ras-dependent transformation phenotypes in human cancer cells.. Proc Natl Acad Sci USA.

[33] Sarma KD, Zhang J, Huang Y, Davidson JG (2006). Amino Acid Esters and Amides for Reductive Amination of Mucochloric Acid: Synthesis of Novel γ-Lactams, Short Peptides and Antiseizure Agent Levetiracetam (Keppra®).. Eur J Org Chem.

[34] Wittchen ES, Aghajanian A, Burridge K (2011). Isoform-specific differences between Rap1A and Rap1B GTPases in the formation of endothelial cell junctions.. Small Gtpases.

[35] Terada K, Mori M (2000). Human DnaJ homologs dj2 and dj3, and bag-1 are positive cochaperones of hsc70.. J Biol Chem.

[36] Kazi A, Carie A, Blaskovich MA, Bucher C, Thai V (2009). Blockade of protein geranylgeranylation inhibits Cdk2-dependent p27Kip1 phosphorylation on Thr187 and accumulates p27Kip1 in the nucleus: implications for breast cancer therapy.. Mol Cell Biol.

[37] Allal C, Pradines A, Hamilton AD, Sebti SM, Favre G (2002). Farnesylated RhoB prevents cell cycle arrest and actin cytoskeleton disruption caused by the geranylgeranyltransferase I inhibitor GGTI-298.. Cell Cycle.

[38] Adnane J, Bizouarn FA, Qian Y, Hamilton AD, Sebti SM (1998). p21(WAF1/CIP1) is upregulated by the geranylgeranyltransferase I inhibitor GGTI-298 through a transforming growth factor beta- and Sp1-responsive element: involvement of the small GTPase rhoA.. Mol Cell Biol.

[39] Olson MF, Paterson HF, Marshall CJ (1998). Signals from Ras and Rho GTPases interact to regulate expression of p21Waf1/Cip1.. Nature.

[40] Allal C, Favre G, Couderc B, Salicio S, Sixou S (2000). RhoA prenylation is required for promotion of cell growth and transformation and cytoskeleton organization but not for induction of serum response element transcription.. J Biol Chem.

[41] Sun J, Qian Y, Chen Z, Marfurt J, Hamilton AD (1999). The geranylgeranyltransferase I inhibitor GGTI-298 induces hypophosphorylation of retinoblastoma and partner switching of cyclin-dependent kinase inhibitors. A potential mechanism for GGTI-298 antitumor activity.. J Biol Chem.

[42] Hirai A, Nakamura S, Noguchi Y, Yasuda T, Kitagawa M (1997). Geranylgeranylated Rho small GTPase(s) are essential for the degradation of p27Kip1 and facilitate the progression from G1 to S phase in growth-stimulated rat FRTL-5 cells.. J Biol Chem.

[43] Hu W, Bellone CJ, Baldassare JJ (1999). RhoA stimulates p27(Kip) degradation through its regulation of cyclin E/CDK2 activity.. J Biol Chem.

[44] Croft DR, Olson MF (2006). The Rho GTPase effector ROCK regulates cyclin A, cyclin D1, and p27Kip1 levels by distinct mechanisms.. Mol Cell Biol.

[45] Joyce PL, Cox AD (2003). Rac1 and Rac3 are targets for geranylgeranyltransferase-I inhibitor-mediated inhibition of signaling, transformation, and membrane ruffling.. Cancer Res.

