# Supplementary figures and images for: Identification and Characterization of Mechanism of Action of P61-E7, a Novel Phosphine Catalysis-Based Inhibitor of Geranylgeranyltransferase-I
Source: PLoS One. 2011 Oct 18;6(10):e26135. doi: 10.1371/journal.pone.0026135 (PMC3196516; doi:10.1371/journal.pone.0026135)

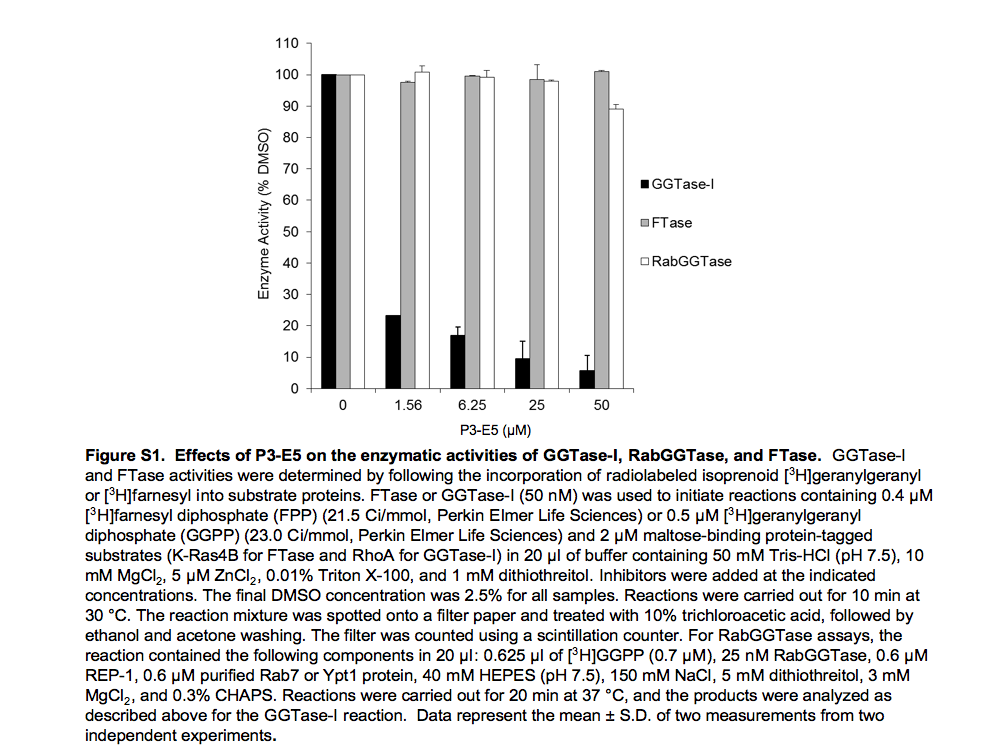

Supplement: Figure S1 — Effects of P3-E5 on the enzymatic activities of GGTase-I, RabGGTase, and FTase. GGTase-I and FTase activities were determined by following the incorporation of radiolabeled isoprenoid [3H]geranylgeranyl or [3H]farnesyl into substrate proteins. FTase or GGTase-I (50 nM) was used to initiate reactions containing 0.4 µM [3H]farnesyl diphosphate (FPP) (21.5 Ci/mmol, Perkin Elmer Life Sciences) or 0.5 µM [3H]geranylgeranyl diphosphate (GGPP) (23.0 Ci/mmol, Perkin Elmer Life Sciences) and 2 µM maltose-binding protein-tagged substrates (K-Ras4B for FTase and RhoA for GGTase-I) in 20 µl of buffer containing 50 mM Tris-HCl (pH 7.5), 10 mM MgCl2, 5 µM ZnCl2, 0.01% Triton X-100, and 1 mM dithiothreitol. Inhibitors were added at the indicated concentrations. The final DMSO concentration was 2.5% for all samples. Reactions were carried out for 10 min at 30°C. The reaction mixture was spotted onto a filter paper and treated with 10% trichloroacetic acid, followed by ethanol and acetone washing. The filter was counted using a scintillation counter. For RabGGTase assays, the reaction contained the following components in 20 µl: 0.625 µl of [3H]GGPP (0.7 µM), 25 nM RabGGTase, 0.6 µM REP-1, 0.6 µM purified Rab7 or Ypt1 protein, 40 mM HEPES (pH 7.5), 150 mM NaCl, 5 mM dithiothreitol, 3 mM MgCl2, and 0.3% CHAPS. Reactions were carried out for 20 min at 37°C, and the products were analyzed as described above for the GGTase-I reaction. Data represent the mean ± S.D. of two measurements from two independent experiments. (TIF) [file pone.0026135.s001.tif]

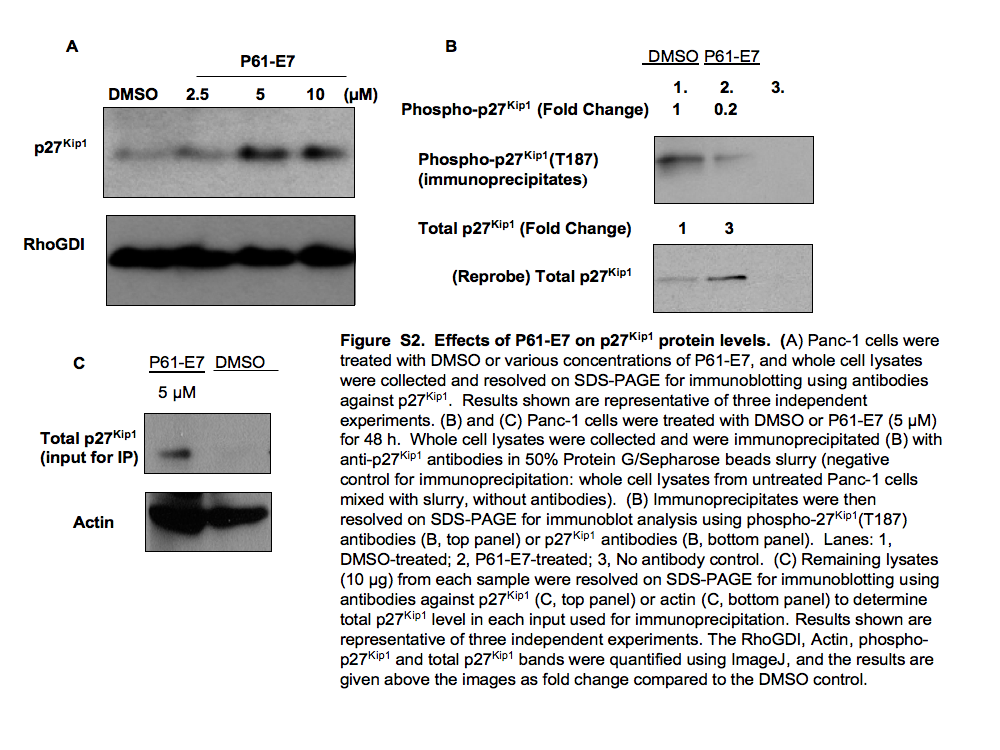

Supplement: Figure S2 — Effects of P61-E7 on p27Kip1 protein levels. (A) Panc-1 cells were treated with DMSO or various concentrations of P61-E7, and whole cell lysates were collected and resolved on SDS-PAGE for immunoblotting using antibodies against p27Kip1. Results shown are representative of three independent experiments. (B) and (C) Panc-1 cells were treated with DMSO or P61-E7 (5 µM) for 48 h. Whole cell lysates were collected and were immunoprecipitated (B) with anti-p27Kip1 antibodies in 50% Protein G/Sepharose beads slurry (negative control for immunoprecipitation: whole cell lysates from untreated Panc-1 cells mixed with slurry, without antibodies). (B) Immunoprecipitates were then resolved on SDS-PAGE for immunoblot analysis using phospho-27Kip1(T187) antibodies (B, top panel) or p27Kip1 antibodies (B, bottom panel). Lanes: 1, DMSO-treated; 2, P61-E7-treated; 3, No antibody control. (C) Remaining lysates (10 µg) from each sample were resolved on SDS-PAGE for immunoblotting using antibodies against p27Kip1 (C, top panel) or actin (C, bottom panel) to determine total p27Kip1 level in each input used for immunoprecipitation. Results shown are representative of three independent experiments. The RhoGDI, Actin, phospho-p27Kip1 and total p27Kip1 bands were quantified using ImageJ, and the results are given above the images as fold change compared to the DMSO control. (TIF) [file pone.0026135.s002.tif]

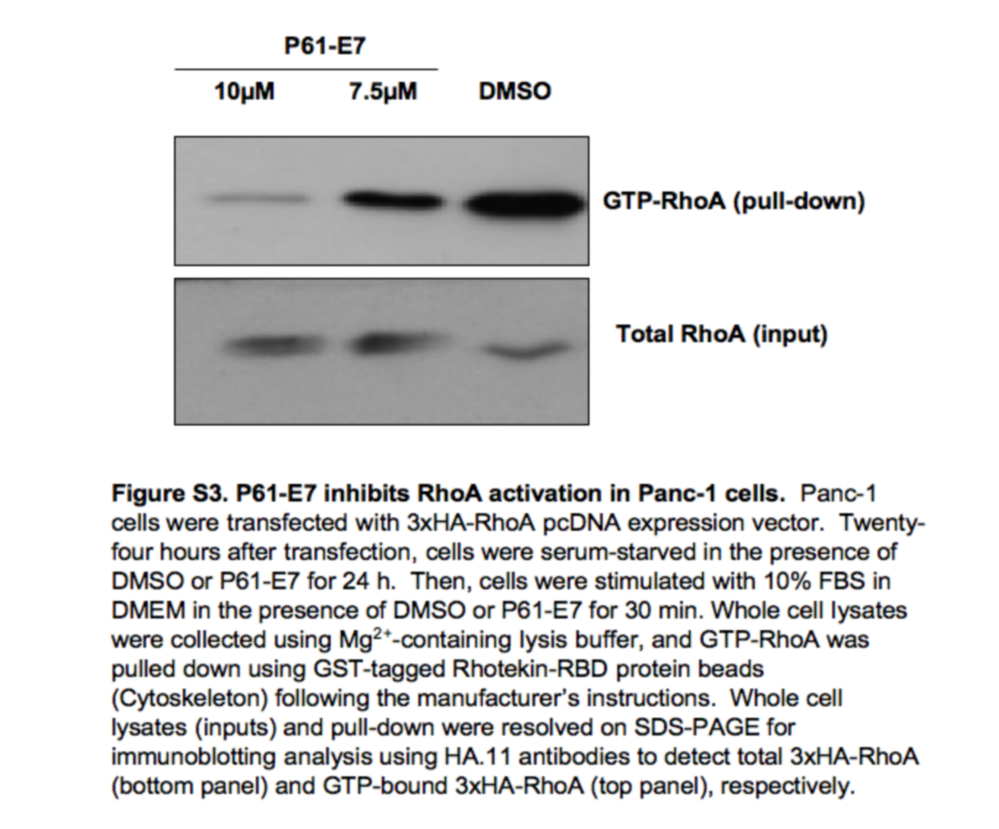

Supplement: Figure S3 — P61-E7 inhibits RhoA activation in Panc-1 cells. Panc-1 cells were transfected with 3xHA-RhoA pcDNA expression vector. Twenty-four hours after transfection, cells were serum-starved in the presence of DMSO or P61-E7 for 24 h. Then, cells were stimulated with 10% FBS in DMEM in the presence of DMSO or P61-E7 for 30 min. Whole cell lysates were collected using Mg2+-containing lysis buffer, and GTP-RhoA was pulled down using GST-tagged Rhotekin-RBD protein beads (Cytoskeleton) following the manufacturer's instructions. Whole cell lysates (inputs) and pull-down were resolved on SDS-PAGE for immunoblotting analysis using HA.11 antibodies to detect total 3xHA-RhoA (bottom panel) and GTP-bound 3xHA-RhoA (top panel), respectively. (TIF) [file pone.0026135.s003.tif]
